# Obesity Risk of Pediatric Central Nervous System Tumor Survivors: A Cross-Sectional Study

**DOI:** 10.3390/nu15102269

**Published:** 2023-05-11

**Authors:** Rebekah L. Wilson, Jacqueline Soja, Alexandra G. Yunker, Hajime Uno, Erin Gordon, Tabitha Cooney, Christina M. Dieli-Conwright

**Affiliations:** 1 Division of Population Sciences, Department of Medical Oncology, Dana-Farber Cancer Institute, Boston, MA 02215, USA; rebekahl_wilson@dfci.harvard.edu (R.L.W.);; 2 Department of Medicine, Harvard Medical School, Boston, MA 02215, USA; 3 Boston Children’s Cancer and Blood Disorders Center, Boston, MA 02215, USA; 4 Department of Nutrition, Harvard T.H. Chan School of Public Health, Boston, MA 02215, USA; 5 Boston Children’s Hospital, Boston, MA 02215, USA

**Keywords:** nutrition status, pediatric central nervous system tumors, obesity risk, young adults, risk stratification

## Abstract

Adult survivors of pediatric central nervous system (CNS) tumors are at the highest risk for morbidity and late mortality among all childhood cancers due to a high burden of chronic conditions, and environmental and lifestyle factors. This study aims to epidemiologically characterize young adult survivors of pediatric CNS tumors using body mass index (BMI) to assess risk factors for obesity. Using a cross-sectional design, young adults (18–39 years) previously treated for pediatric CNS tumors and followed in a survivorship clinic during 2016–2021 were examined. Demographic, BMI, and diagnosis information were extracted from medical records of the most recent clinic visit. Data were assessed using a two-sample t-test, Fisher’s exact test, and multivariable logistical regression. 198 survivors (53% female, 84.3% White) with a BMI status of underweight (4.0%), healthy weight (40.9%), overweight (26.8%), obesity (20.2%), and severe obesity (8.1%) were examined. Male sex (OR, 2.414; 95% CI, 1.321 to 4.414), older age at follow-up (OR, 1.103; 95% CI, 1.037 to 1.173), and craniopharyngioma diagnosis (OR, 5.764; 95% CI, 1.197 to 27.751) were identified as significant (*p* < 0.05) obesity-related (≥25.0 kg/m^2^) risk factors. The majority of patients were overweight or obese. As such, universal screening efforts with more precise determinants of body composition than BMI, risk stratification, and targeted lifestyle interventions are warranted during survivorship care.

## 1. Introduction

Central nervous system (CNS) tumors are the second most common childhood cancer with 75% of children diagnosed surviving ≥5 years [1]. With this high long-term survival rate comes a need for clinical care with a focus on late effects, which can be just as detrimental to a survivor’s health as the original cancer diagnosis. Specifically, among pediatric cancer survivors, pediatric CNS tumor survivors are at the highest risk for new chronic medical conditions (e.g., cardiovascular disease, endocrinopathies), poor health-related quality of life, and late mortality, with a cumulative mortality rate of more than 27% by 45 years old [2,3,4,5,6]. Obesity, which has a prevalence of 28.8–42.0% in pediatric CNS tumor survivors [7,8,9], is a known modifiable risk factor for secondary malignancies and higher cancer relapse in the pediatric cancer population [9,10,11]. Additionally, survivors of CNS tumors are at increased risk of obesity development, potentially as a result of hypothalamic insult, metabolic changes, or reduced physical activity levels as a result of the tumor and/or its treatment [12,13,14]. At the other end of the spectrum, pediatric cancer survivors with an underweight status have an increased likelihood of reporting adverse health and major medical conditions, which may also contribute to early mortality [8]. 

Monitoring of the nutritional status of adult survivors of pediatric CNS tumors is encouraged within the survivorship guidelines [15]; however, identification of key risk factors of poor nutritional status among pediatric CNS tumor survivors that need the most attention, how nutritional status should be assessed, and updated comparison studies with the general population where ongoing societal increases in obesity levels continue to trend [16] are not well examined. The literature examining the nutritional status within the pediatric cancer population is sparse with limited work completed within the past 5–10 years and the majority of the research conducted in hematological cancers [13,17]. A systematic review examining the prevalence of malnutrition in pediatric cancer patients identified 29 studies targeting hematological cancers, 13 examining solid tumors, two on brain tumors, eight targeting those with International Classification of Childhood Cancer (3rd edition), and six including a mixed cohort [13]. As such, the CNS tumor survivor population is drastically underrepresented within malnutrition-related research, despite obesity being a known late effect within this population.

As adults, more than 45% of survivors of pediatric CNS tumors are likely to experience at least one or more poor health outcomes (e.g., poor general health, adverse mental health, functional impairment, activity limitations) compared to less than 20% of non-cancer siblings [18]. Furthermore, poor health outcomes are further exacerbated by the presence of malnutrition, either underweight or obesity, a risk factor that can be modified [13,18]. As such, to know who, if, how, and when to intervene with regard to improving nutritional status, it is important to understand the current scope of malnutrition within the pediatric CNS tumor survivor population. While the limitations of BMI have become increasingly known and scrutinized for its inability to distinguish between body composition (i.e., fat mass vs. muscle mass), differences amongst ethnic populations, and inability to provide any indicator of metabolic health, it continues to be a simple screening tool to help identify those at risk for potential health and/or nutritional complications. Additionally, current diagnostic criteria for malnutrition (both under- and over-nutrition) continue to use BMI as an indicator of overall nutrition status. Therefore, the purpose of this observational study was to epidemiologically characterize young adult survivors of pediatric CNS tumors using BMI to assess risk factors of obesity. 

## 2. Materials and Methods

### 2.1. Study Design and Population

Data collected for this retrospective, cross-sectional study were obtained from medical records of young adults seen through the Stop & Shop Family Pediatric Neuro-Oncology Outcomes Clinic at Dana-Farber/Boston Children’s Cancer and Blood Disorders Center. The clinic provides multi-disciplinary, long-term follow-up to survivors of pediatric CNS tumors through young adulthood. Patients were eligible for this analysis if they were aged 18–39 years at the time of their most recent visit, previously diagnosed with a CNS tumor at 18 years or younger, and were seen in the survivorship clinic between 2016 and 2021. Due to the minimal risk of the study to patients, a consent waiver was granted. 

### 2.2. Data Collection

Data obtained from medical records included: (1) height (cm) and weight (kg) data from most recent survivorship appointment, these were used to calculate BMI, (2) age at diagnosis and follow-up appointment, (3) sex (male, female), (4) race (White, Black, Asian, other or multiple races) and ethnicity (Hispanic/Latino yes/no), (5) insurance (Medicaid/Mass health, private, other), (6) tumor histology (low-grade glioma, embryonal, craniopharyngioma, other e.g., ependymoma, choroid plexus tumor, germ cell tumor), (7) tumor location (posterior fossa, hypothalamus/optic pathway, supratentorial, cervicomedullary, spinal cord), (8) treatment (surgery, cranial radiotherapy exposure, chemotherapy), (9) presence of neurodevelopmental and/or endocrine disorder, and (10) current stimulant medication use (methylphenidate, amphetamine/dextroamphetamine). 

### 2.3. Definitions

*Age at diagnosis and follow-up.* Age at diagnosis was taken from the date of magnetic resonance imaging showing tumor presence. If an exact date was not available, then the first of the month was entered. Age at follow-up was taken from the most recent visit to the survivorship clinic between 2016 and 2021 when both weight and height variables were available.

*Underweight, healthy weight, overweight, obesity, and severe obesity.* BMI was calculated by dividing weight (kg) by height squared (kg/m^2^) that was recorded at the most recent survivorship clinic appointment. Underweight was defined as a BMI < 18.5 kg/m^2^, healthy weight 18.5–24.9 kg/m^2^, overweight 25.0–29.9 kg/m^2^, obesity (class I) 30.0–34.9 kg/m^2^, and severe obesity (combined class II and III) ≥ 35.0 kg/m^2^ [19]. 

*Treatment.* Receipt of surgery included those who had a biopsy, partial/subtotal resection, and/or a total resection. Those who only had a shunt procedure were not classified as having had a tumor-related surgery. Only those patients who received cranial radiation were identified as being exposed to radiotherapy. Finally, any persons who received chemotherapy were identified as exposed to chemotherapy.

*Endocrine disorder.* An endocrine disorder was defined as any condition relating to the hypothalamic-pituitary system including growth hormone deficiency, thyroid stimulating hormone deficiency, adrenocorticotropic hormone deficiency, gonadotropic releasing hormone deficiency, diabetes insipidus, central precocious puberty, disorders of the thyroid and gonads. 

*Neurodevelopment disorder.* A neurodevelopment disorder was defined as any condition that influences brain function and alters neurological development including autism, attention deficit hyperactivity disorder, speech, motor, and learning disorders, and intellectual disability. 

*Stimulant use.* Stimulant use was defined by whether patients were currently taking methylphenidate or amphetamine/dextroamphetamine at the time of their most recent survivorship clinic visit.

### 2.4. Statistical Analysis

Statistical analyses were conducted using IBM SPSS version 28 (SPSS Inc., IBM, Armonk, NY, USA). The normality of distribution was assessed using the Kolmogorov–Smirnov test. Data are presented as mean ± standard deviation (SD), median and interquartile range [IQR], or number (percentage). The two-sample *t*-test, or the Wilcoxon rank sum test for the non-normally distributed variables, was used to assess between-group differences for continuous variables and Fisher’s exact test for categorical data. Forward stepwise logistic regression analysis was used to identify risk factors for overweight and obesity diagnosis. Patients that were underweight or healthy weight were grouped together as the reference group and compared to those classed as overweight, obese, and severely obese given that a BMI ≥ 25.0 kg/m^2^ is associated with a higher risk of mortality [20]. The result is presented as odd ratios (ORs) and 95% confidence intervals (CIs). Independent variables to be included in the forward stepwise variable selection procedure were selected based on the significance of the bivariate association between the variables and the weight group, in addition to variables deemed clinically relevant (e.g., an endocrine disorder, location, and histology of tumor). In an exploratory analysis, due to a small number of underweight survivors, only bivariate associations were examined for differences in the variables between underweight and those not underweight. Tests were two-tailed and statistical significance was set at *p* < 0.05. 

## 3. Results

### 3.1. Patient Characteristics

**Figure 1:**
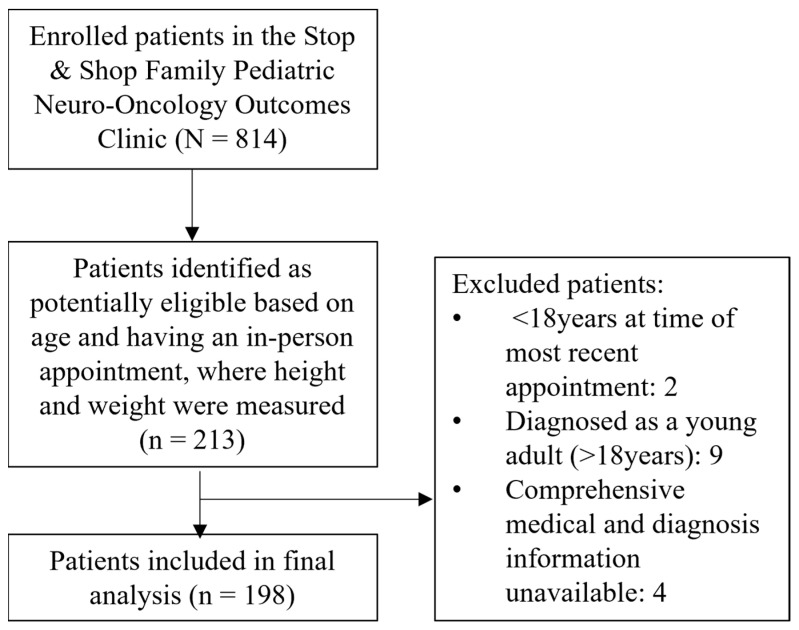
CONSORT diagram.

We identified 198 (53% female) survivors of childhood CNS tumors that met the inclusion criteria (Figure 1). The cohort was separated into five BMI categories where 4% were identified as underweight, 40.9% as healthy weight, 26.8% as overweight, 20.3% as obese, and 8.1% as severely obese (Table 1). Overall, patients predominantly identified as White (84.3%), non-Hispanic/Latino (66.7%), and were diagnosed at a median age of 8 (4–12) years. At the time of the most recent survivorship clinic appointment, patients were a median age of 24 (20–28) years and had been followed for a mean of 16.5 ± 6.4 years. Tumors were commonly located in the posterior fossa (39.4%), with low-grade glioma being the most prevalent primary tumor histology (50.5%). The majority of patients received surgery (90.4%) with just over half of patients exposed to chemotherapy (51.5%) and cranial radiotherapy (52.5%). Finally, 41.9% were identified as having an endocrine disorder, 23.2% as a neurodevelopmental disorder, and 12.6% were taking stimulants at the time of their most recent survivorship clinic appointment.

### 3.2. Risk of Overweight and Obesity at Follow-Up

**Figure 2:**
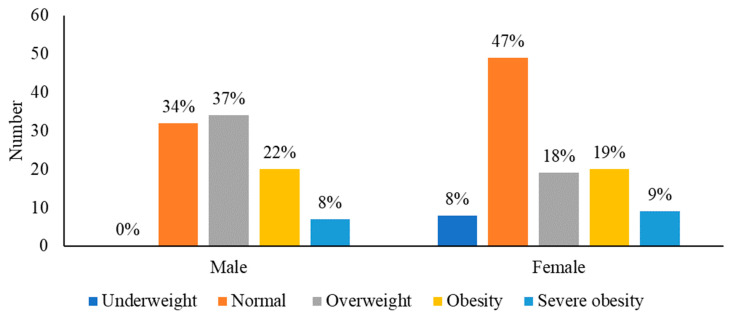
Distribution of sex across the five BMI groups.

At the most recent survivorship appointment, 109 (55.1%) survivors had overweight (n = 53, 26.8%), obesity (n = 40, 20.2%), or severe obesity (n = 16, 8.1%), after a mean follow-up time of 16.8 ± 5.6 years, 17.9 ± 7.3 years, and 19.7 ± 6.2 years respectively. A significant difference in the prevalence of patients with overweight and obesity was found between females and males (*p* = 0.006) (Figure 2). On multivariate analyses, male sex (OR, 2.414; 95% CI, 1.321 to 4.414), older age at follow-up (OR, 1.103; 95% CI, 1.037 to 1.173), and diagnosis of craniopharyngioma (OR, 5.764; 95% CI, 1.197 to 27.751) were associated with overweight or obesity at last follow-up (Table 2). Twelve (85.7%) of the craniopharyngioma survivors had overweight (n = 2, 14.3%), obesity (n = 5, 35.7%), or severe obesity (n = 5, 35.7%) at the last follow-up. In sub-hoc analysis, which excluded those with a craniopharyngioma diagnosis (n = 184), male sex (OR, 2.312; 95% CI, 1.256 to 4.256, *p* = 0.007) and older age at follow-up (OR, 1.101; 95% CI, 1.034 to 1.172, *p* = 0.003) were still significant risk factors for overweight/obesity.

### 3.3. Underweight at Follow-Up

At the most recent follow-up, 8 (4.0%) survivors were underweight, after a mean follow-up of 14.2 ± 6.3 years. A significant difference in underweight prevalence between females and males was found (*p* = 0.007) (Figure 2). On bivariate analysis, female sex (100.0% vs. 51.1%, *p* = 0.007) and non-white race (50.0% vs. 14.2%, *p* = 0.022) were significantly associated with underweight status at follow-up when compared to survivors with healthy weight, overweight, obesity, or severe obesity. One (7.1%) had a tumor in the hypothalamic/optic pathway, which was a craniopharyngioma. Underweight survivors received cranial radiotherapy less frequently when compared with survivors with healthy weight, overweight, obesity, or severe obesity (12.5% vs. 54.2%, *p* = 0.028) (Table 1). 

## 4. Discussion

We evaluated the BMI status of young adult survivors of pediatric CNS tumors followed in a pediatric neuro-oncology survivorship clinic. There were three important findings: (1) over half (55.1%) of the population analyzed were identified to have overweight, obesity, or severe obesity, (2) those who were male sex, older age at follow-up, and had a craniopharyngioma diagnosis were more likely to have overweight, obesity, or severe obesity, and (3) female sex, non-white race, and exposure to radiotherapy were more likely among patients with an underweight status, compared with non-underweight. 

Presence of obesity in childhood cancer survivors is associated with an increased risk of relapse, obesity-related comorbidity development (e.g., type two diabetes, metabolic syndrome), obesity-related cancer development (e.g., colorectal, kidney) as well as decreased overall survival [11]. Within the examined cohort, 55.1% were overweight, obese, or severely obese. Compared to other studies examining survivors of pediatric CNS tumors with shorter follow-up durations, the present study found a higher prevalence of obesity rates (55.1% vs. 28.8–42.0%) [7,8,9]. Of note, Wilson et al. [21] examined 158 survivors of pediatric CNS tumors with an older median age at follow-up (32.4 years) and reported an overweight and obese prevalence of 66.4%. Given the present analysis determined age at a follow-up appointment to be a significant risk factor for obesity presence, it is likely the rates of overweight/obesity continue to climb throughout survivorship years, similar to the general population [14,22]. Furthermore, the proportion of obesity (28.3%; BMI > 30 kg/m^2^) in our cohort is comparable to the general U.S. young adult population of 32.7% (20–25 years) (assessed 1976–2018) [16]. These findings would suggest similar rates of obesity-related cardiometabolic comorbidities between age-matched pediatric CNS tumor survivors and non-cancer controls; however, this is not the case. For example, significant relative risks between 2.0 and 359.7 have been reported for survivors of pediatric CNS tumors in developing endocrine and cardiovascular-related conditions within five years post-diagnosis when compared to siblings [23]. This discrepancy in cardiometabolic comorbidities, but no difference in BMI status, would suggest that the tumor and its treatment may impact the adiposity levels and metabolic function of survivors. Such impact has been highlighted in a systematic review by Wang et al. [24] where despite similar BMI distributions, body fat percent was 4.1% greater among survivors of CNS tumors compared to non-cancer controls. While BMI is associated with adiposity and metabolic dysregulation at the population level, it does not always reflect accurate obesity or generalizable nutrition status at the individual level. Therefore, a further examination into the body composition and metabolic health of survivors of pediatric CNS tumors is required to better understand the potential mechanisms involved in their increased risk of obesity-driven comorbidities [24].

Within this analysis we found male sex to be a significant risk factor associated with overweight, obesity, or severe obesity during survivorship. This is an interesting finding as the majority of the previous research has identified females to be at a higher risk [11,25,26,27]. A systematic review comparing the prevalence of overweight and obesity between survivors of pediatric brain tumors and non-cancer controls reported male cancer survivors to be at higher odds of an overweight status compared to female cancer survivors, but the risk was similar between both sexes when examining an obesity status [24]. Similar to the systematic review, our study did not restrict tumor type, unlike Lek, Prentice [25] who only examined suprasellar brain tumors, which may partially explain the discrepancy in findings. Additionally, our study exclusively targeted survivors ≥ 18 years of age, as such pubertal state in previous studies may also explain sex-associated risk discrepancies. Mechanistic insight into why one sex over the other may be more vulnerable to developing obesity during survivorship is unclear. However, while not in line with the findings of the current analysis, Lek et al. [25] proposed that hyperleptinemia induced by injury to the hypothalamus may play a role in that females typically have higher leptin levels than males for the same degree of fat mass and as such females may develop leptin resistance and have a greater loss of appetite suppression [28]. Given the conflicting evidence, further analysis into whether females or males are at a higher risk of obesity as a young adult survivor of a CNS tumor, as well as mechanistic insight into why this may be, is needed.

Older age at most recent survivorship appointment and a craniopharyngioma diagnosis were also identified as obesity-related risk factors, which is in line with age-related trends in the general population [29,30] and previous pediatric CNS tumor studies [9,11,14,17,22]. The location of the craniopharyngioma tumor and damage to the hypothalamus because of the tumor and cancer-related therapies can lead to the development of “hypothalamic obesity,” a syndrome in which lifestyle interventions are rarely effective [31]. Nonetheless, physical activity may still be critical in obesity management, where a lack of physical activity has also been noted as a potential key component of obesity development among patients with craniopharyngioma [32]. Further analysis is required to identify how much physical inactivity contributes to obesity development, in addition to why physical activity is lower among survivors of both craniopharyngioma and other CNS tumors. Refined understanding will assist in targeted interventions to manage and modify obesity status. 

An underweight status is uncommon among survivors of pediatric CNS tumors in Western countries [9,33]. This is reflected in the current analysis with only 4.0% of the assessed population with an underweight status. However, being underweight is still associated with comorbidity development (e.g., frailty), or a higher risk of mortality [11,34]. Consequently, we did a bi-variate analysis between those with underweight versus not underweight statuses. Because of the small sample size of survivors with underweight (n = 8), caution is warranted with this analysis; however, female sex, non-white race, and no exposure to radiotherapy are potential candidates as risk factors for developing underweight as a young adult survivor of pediatric CNS tumors. 

This study has several strengths. The cohort analyzed represents a typical distribution of tumor types, locations, and treatments from a unique clinical setup that is dedicated to enhancing survivorship quality among young adult survivors of CNS tumors. Additionally, compared to similar studies [9,25,35], our study describes the longest follow-up time (16.5 ± 6.4 years) providing a better epidemiological description of BMI status in young adult survivors of pediatric CNS tumors. However, there are several limitations. This was a cross-sectional study of a single institution analysis with the majority of participants identifying as White, therefore, the generalizability of this study to populations not racialized as White is unclear. Additionally, while our study targeted survivors, we need to consider survivor bias where those who were underweight may have died shortly after diagnosis. While BMI is often used as an easy, inexpensive variable to gauge the nutrition status of a patient or population, the distribution of body composition (i.e., fat and muscle mass) provides more accurate information regarding metabolic health and mechanistic understanding of obesity development within the CNS tumor population. Additionally, CNS tumor patients are known to be at risk of short stature. BMI relies on height across a normal distribution, which may result in the misclassification of obesity, and further emphasizes that body composition assessment (e.g., fat and muscle tissue) may be a useful measure when considering obesity management strategies [26,36]. 

## 5. Conclusions

Within the current analysis, the majority of patients were overweight, obese, or severely obese, which is comparable to the non-cancer young adult U.S. population. Our refined understanding of the impact of disease and treatment exposure in this population will allow for more strategic identification of potential survivors in need of obesity management. Within this analysis, we identified male sex, age at follow-up, and craniopharyngioma diagnosis as key risk factors for obesity development. While these may help guide clinicians to identify at-risk survivors, the ideal intervention to prevent or modify obesity is still unclear in this population. While some forms of obesity within the CNS tumor population may respond to lifestyle-based interventions, those with hypothalamic obesity may not respond to such interventions [11,22]. To date, there are no combined nutrition and exercise-based interventions within the pediatric CNS tumor population, either on or off treatment [37]. Future studies are required to examine the benefit of such interventions to establish the best prescription to manage not only obesity status but adiposity and metabolic function, among long-term survivors of CNS tumors. 

## Figures and Tables

**Table 1:** Characteristics of young adult survivors of pediatric CNS tumors with a bivariate analysis comparing overweight, obesity, and severe obesity versus those without this status; similarly comparing underweight status with those without this status.

Characteristic	Total (N = 198; 100%)	Underweight (n = 8; 4.0%)	Healthy weight (n = 81; 40.9%)	Overweight (n = 53; 26.8%)	Obesity (n = 40; 20.2%)	Severe obesity (n = 16; 8.1%)	*p*-Value	
							Overweight or Obese vs. Not	Underweight vs. Not
Sex N (%)								
Male	93 (47.0)	-	32 (39.5)	34 (64.2)	20 (50.0)	7 (43.8)	***0.006***	***0.007***
Female	105 (53.0)	8 (100.0)	49 (60.5)	19 (35.8)	20 (50.0)	9 (56.3)		
Age Median [IQR]								
At diagnosis, years	8 [4.0–12.0]	6 [3.5–14.5]	8 [5.0–13.0]	8 [3.5–12.0]	10 [5.0–12.8]	5.5 [3.3–9.0]	0.860	0.743
At follow-up, years	24.0 [20.0–28.0]	20.5 [19.3–25.5]	23.0 [20.0–27.0]	24.0 [20.0–28.0]	27.0 [20.3–32.8]	25.0 [23.3–31.8]	***0.002***	0.181
Follow-up time, years (mean ± SD)	16.5 ± 6.4	14.2 ± 6.3	15.1 ± 6.2	16.8 ± 5.6	17.9 ± 7.3	19.7 ± 6.2	***0.005***	0.323
Race N (%)								
White	167 (84.3)	4 (50.0)	70 (86.4)	47 (88.7)	31 (77.5)	15 (93.8)	0.698	***0.022***
Black/African American	8 (4.0)	3 (37.5)	3 (3.7)	1 (1.9)	1 (2.5)	-		
Asian	2 (1.0)	-	-	2 (3.8)	-	-		
Other or multiple races	7 (3.5)	1 (12.5)	2 (2.5)	-	4 (10.0)	-		
Unknown	14 (7.1)	-	6 (7.4)	3 (5.7)	4 (10.0)	1 (6.3)		
Ethnicity N (%)								
Hispanic/Latino	14 (7.1)	-	6 (7.4)	2 (3.8)	4 (10.0)	2 (12.5)	1.000	0.229
Not Hispanic/Latino	132 (66.7)	8 (100.0)	46 (56.8)	37 (69.8)	28 (70.0)	13 (81.3)		
Unknown	52 (26.3)	-	29 (35.8)	14 (26.4)	8 (20.0)	1 (6.3)		
Insurance N (%)								
Medicaid/Mass Health	78 (39.4)	4 (50.0)	24 (29.6)	21 (39.6)	21 (52.5)	8 (50.0)	0.109	0.760
Private	116 (58.6)	4 (50.0)	55 (67.9)	31 (58.5)	18 (45.0)	8 (50.0)		
Other	4 (2.0)	-	2 (2.5)	1 (1.9)	1 (2.5)	-		
Tumor histology at primary diagnosis N (%)								
Low-grade glioma	100 (50.5)	6 (75.0)	44 (54.3)	25 (47.2)	19 (47.5)	6 (37.5)	0.087	0.054
Embryonal tumor	40 (20.2)	-	18 (22.2)	11 (20.8)	9 (22.5)	2 (12.5)		
Craniopharyngioma	14 (7.1)	1 (12.5)	1 (1.2)	2 (3.8)	5 (12.5)	5 (31.3)		
Other	44 (22.2)	1 (12.5)	18 (22.2)	15 (28.3)	7 (17.5)	3 (18.8)		
Primary tumor location N (%)								
Posterior fossa	78 (39.4)	3 (37.5)	33 (40.7)	22 (41.5)	16 (40.0)	4 (25.0)	0.295	0.054
Hypothalamus/optic pathway	44 (22.2)	1 (12.5)	13 (16.0)	9 (17.0)	13 (32.5)	8 (50.0)		
Supratentorial	62 (31.3)	2 (25.0)	30 (37.0)	16 (30.2)	11 (27.5)	3 (18.8)		
Cervicomedullary	5 (2.5)	2 (25.0)	1 (1.2)	1 (1.9)	-	1 (6.3)		
Spinal cord	8 (4.0)	-	4 (4.9)	4 (7.5)	-	-		
Tumor treatment N (%)								
Surgery	179 (90.4)	7 (87.5)	74 (91.4)	48 (90.6)	36 (90.0)	14 (87.5)	1.000	0.561
Chemotherapy	102 (51.5)	2 (25.0)	48 (59.3)	25 (47.2)	19 (47.5)	8 (50.0)	0.255	0.160
Radiotherapy	104 (52.5)	1 (12.5)	36 (44.4)	29 (54.7)	26 (65.0)	12 (75.0)	***0.007***	***0.028***
Presence of neurodevelopmental disorder N (%)	46 (23.2)	1 (12.5)	16 (19.8)	14 (26.4)	12 (30.0)	3 (18.8)	0.239	0.684
Presence of endocrine disorder N (%)	83 (41.9)	2 (25.0)	29 (35.8)	21 (39.6)	22 (55.0)	9 (56.3)	0.083	0.472
Stimulant use N (%)	25 (12.6)	1 (12.5)	12 (14.8)	7 (13.2)	3 (7.5)	2 (12.5)	0.521	1.000

**Table 2:** Regression analysis assessing risk factors for developing overweight, obesity, or severe obesity during survivorship care.

Variable	Odds Ratio	95% CI	*p* Value
Males	2.414	1.321–4.414	***0.004***
Age at follow up	1.103	1.037–1.173	***0.002***
Craniopharyngioma	5.764	1.197–27.751	***0.029***

## Data Availability

Available on request.
